# Dietary protein supplementation results in molecular and cellular changes related to T helper type 2 immunity in the lung and small intestine in lactating rats re-infected with *Nippostrongylus brasiliensis*

**DOI:** 10.1017/S0031182021001876

**Published:** 2022-03

**Authors:** Aya Masuda, Judith E. Allen, Jos G. M. Houdijk, Spiridoula Athanasiadou

**Affiliations:** 1 Animal & Veterinary Sciences, SRUC, Roslin Institute Building, Easter Bush, UK; 2 Division of Infection, Immunity and Respiratory Medicine, School of Biological Sciences, University of Manchester, Manchester, UK

**Keywords:** dietary protein, gastrointestinal nematodes, lactation, *Nippostrongylus brasiliensis*, periparturient relaxation of immunity, T helper type 2 immunity

## Abstract

Acquired immunity to gastrointestinal nematodes reduces during late pregnancy and lactation which is known as periparturient relaxation of immunity (PPRI). Protein supplementation reduces the degree of PPRI in a rat model re-infected with *Nippostrongylus brasiliensis*, but the underlying molecular mechanisms have yet to be elucidated. Here, we hypothesized that protein supplementation will enhance T helper type 2 immunity (Th2) in the lung and small intestine. Nulliparous Sprague-Dawley rats were given a primary infection of *N. brasiliensis* prior to mating and restrictedly fed diets with either low protein (LP) or high protein (HP) during pregnancy and lactation. Dams were secondary infected with *N. brasiliensis* on day 2 post-parturition, and histology and gene expression were analysed for tissue samples collected at days 5, 8 and 11. Genes related to Th2 immunity in the lung, *Retnla*, *Il13* and *Mmp12*, and in the intestine, *Retnlb*, were upregulated in HP dams compared to LP dams, which indicates the effect of dietary protein on Th2 immunity. HP dams also had increased splenic CD68^+^ macrophage populations compared to LP dams following secondary infection, suggesting enhanced immunity at a cellular level. Our data assist to define strategic utilization of nutrient supply in mammals undergoing reproductive and lactational efforts.

## Graphical abstract

**Figure:**
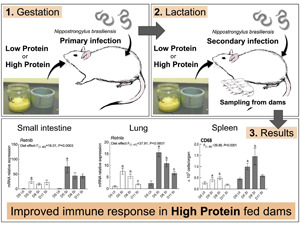

## Introduction

Periparturient relaxation of immunity (PPRI) in mammals, where the expression of acquired immunity reduces during pregnancy and lactation, has been shown to have a nutritional basis. Studies using the *Nippostrongylus brasiliensis* re-infected lactating rat model have repeatedly demonstrated that at times of protein scarcity, dietary protein supplementation significantly reduces fecal nematode egg counts (FEC) and intestinal worm burdens during PPRI (Houdijk *et al*., 2003, 2005). This effect of protein supplementation on reducing the degree of PPRI is further supported by an increase in plasma IgG concentration, in the number of intestinal mucosal mast cells, eosinophils and goblet cells (Jones *et al*., 2009, 2011). It is clear that dietary protein supplementation improves the host's immune response against nematode infection during pregnancy and lactation, but the underlying mechanisms that regulate these responses have yet to be elucidated.

We have previously demonstrated that protein supplementation in periparturient rats resulted in changes in gene expression patterns associated with increased cell turnover and accelerated onset of immune response in the small intestine, contributing to the establishment of an anti-inflammatory environment (Athanasiadou *et al*., 2011; Athanasiadou, 2012). In the small intestine, goblet cell proliferation and mucus secretion are also essential effector mechanisms for effective *N. brasiliensis* expulsion from the host (Miller and Nawa, 1979; Khan *et al*., 2001). However, it is yet unclear whether protein supplementation at times of protein scarcity, may influence the expression of genes related to goblet cells and mucus production, which may have an added impact on parasite survival. Prior to their emergence in the small intestine, *N. brasiliensis* larvae migrate *via* the lung, where the damage caused by the larvae primes protective immunity with enhanced Th2 immune response (Harvie *et al*., 2010; Thawer *et al*., 2014). The Th2 cytokines, IL-4 and/or IL-13, upregulate key molecules involved in repairing the nematode-induced lung injury, including resistin-like molecule alpha (RELM*α*; *Retnla*) and arginase 1 (*Arg1*) (Chen *et al*., 2012; Sutherland *et al*., 2018). Supplementing animals with additional protein may enhance such immune responses and accelerate the process of injury repair in the lung.

Migration of *N. brasiliensis* larvae *via* the lung to the small intestine elicits systemic immune response through the lymphatic system, and T cell and B cell populations from the lymphoid organs are crucial in resolving the infection (Horsnell *et al*., 2013; Mishra *et al*., 2014). Studies suggest that protein malnutrition induces structural changes in the lymphoid organs, such as the thymus and the spleen, resulting in an impaired development of lymphocytes and consequently to diminished immune responses (Savino, 2002; Mello *et al*., 2014). Therefore, the effect of protein supplementation on the lymphoid organs was also examined, because of their likely involvement during the migration of *N. brasiliensis*.

In this study, we hypothesized that under conditions of protein scarcity, protein supplementation mediates local immune responses not only in the small intestine but also in the lung. In particular, we examined whether dietary protein supplementation (1) up-regulates goblet cell-associated transcriptional activity in the small intestine and (2) drives Th2 immune response in the lung in a host undergoing PPRI. Such responses in the lung and the intestine were evaluated together with shifts in T cell, B cell and macrophage populations both in the spleen and in the mesenteric lymph nodes (MLN).

## Materials and methods

### Experimental animals

A total of 58 nulliparous Sprague-Dawley rats, 10–11 weeks old on arrival, were used (Charles River UK Ltd, UK). Rats were housed individually in solid bottom cages, with fresh sawdust provided weekly. For mating purposes, each female was placed in a wire-bottomed cage with a proven male breeder overnight and mating was confirmed through the presence of the vaginal plug. Plastic bubble wrapping material was provided the day before the expected parturition date for nesting. The parturition date was defined as the morning when parturition was observed to have finished and described as day 0.

### Infection protocol

Rats were infected subcutaneously in the hind limb with 1600 L3 of *N. brasiliensis* in 0.5 mL sterile PBS according to a previously established protocol (Houdijk *et al*., 2003). Fifty-two rats were given a primary infection of 1600 L3 subcutaneously in the hind limb 14 days prior to mating, whereas 6 rats were sham infected with 0.5 mL sterilized PBS, defined as uninfected (UI) controls. Feces collection and FEC to confirm the primary infection were performed as described previously (Christie and Jackson, 1982; Houdijk *et al*., 2003). Fifty-two rats received a secondary infection (SI) with 1600 L3 on day 2 of the experiment. At the same time, the six UI controls received a sham-infection with 0.5 mL sterilized PBS. Thus, our infection protocol resulted in the formation of two levels of parasitism (UI, uninfected and SI, secondary infected group).

### Feeding protocol

Following the primary infection and until mating was confirmed, rats were fed standard rat chow *ad libitum* [Standard RM3, 202 g digestible crude protein (CP) and 12.2 MJ digestible energy per kg dry matter (DM), Special Diets Services, UK]. Once mating was confirmed (day −22) all rats were fed the initial gestation diet (IGD) containing 200 g CP per kg DM *ad libitum* during early gestation (until day −12). During late gestation (day −11 until day 0), rats were offered one of two iso-energetic experimental diets *ad libitum*, formulated to supply either 60 g CP per kg DM (low protein; LP) or 200 g CP per kg DM (high protein; HP), with treatment groups balanced for body weight at day −22. From parturition (day 0) onwards, LP and HP dams continued to be fed a low and high protein formulated to supply 100 or 300 g CP per kg DM, respectively. Lactation diets had higher CP content compared to gestation diets to meet the demand for the lactating effort (Jessop, 1997). Rats had free access to fresh water throughout the experiment. Composition and chemical analysis of all diets are provided in Table 1.

**Table 1.** Diet composition and analysed chemical composition of the experimental diets during gestation and lactation

	Phase				
	Gestation			Lactation	
	IGD	LP	HP	LP	HP
Ingredients (g kg^−1^ fresh)					
Casein + methionin^a^	217	62	218	103	309
Casein (Bacarel and Co. Ltd, UK)	215	62	216	102	306
Methionin (Sigma, UK)	2	1	2	1	3
Starch + sucrose^b^	376	487	331	515	308
Starch (Sigma, UK)	251	325	221	343	206
Sucrose	125	162	110	172	103
Corn oil	165	208	208	141	141
Vitamin (Special Diet Services, UK)^c^	47	47	47	47	47
Mineral (Special Diet Services, UK)^d^	47	47	47	47	47
Cornflour	46	46	46	46	46
Choline (Sigma, UK)	7	7	7	7	7
Lecithin (Fischer Scientific, UK)	2	2	2	2	2
Alphacel (MP Biomedicals, USA)	94	94	94	94	94
Chemical analysis					
Dry matter (g kg^−1^ fresh)	639.2	764.3	640.3	714.8	627.5
Crude Protein (g kg^−1^ DM)	204	62.5	155	114	270
Ash (g kg^−1^ DM)	38.2	37.1	38.6	40.4	39.9
ADF (Ashed g kg^−1^ DM)	87.1	79.1	83.3	77.3	88.5
Ether extract (g kg^−1^ DM)	168	212	209	146	147
Gross energy (MJ kg^−1^ DM)	21.1	21.1	22.2	19.8	21.3
Total sugars (g kg^−1^ DM)	96.0	131	80.3	145	82.4
Starch (g kg^−1^ DM)	264	327	248	333	237

DM, dry matter; ADF, acid detergent fibre.

a Difference in protein level among each diet was achieved by adjusting the ingredients in the shaded area.

b Protein includes casein and DL-Methionine, carbohydrate includes starch, sucrose and cellulose.

c Supplied the following per kilogram of diet: thiamine hydrochloride, 0.600 g; riboflavin, 0.600 g; pyridoxine hydrochloride, 0.700 g; niacin, 3 g; D-calcium pantothenate, 1.6 g; folic acid, 0.200 g; biotin, 20 g; vitamin B_12_ (0.1% TRIT), 1 g; vitamin A palmitate (250 000 U g^−1^), 1.6 g; *α*-tocopherol (250 U g^−1^), 20 g; vitamin D_3_ (400 000 IU g^−1^), 0.250 g; menadione, 5 mg.

d Supplied the following per 10 kg of diet: calcium carbonate, 3570 g; monopotassium phosphate, 1960 g; potassium citrate monohydrate, 707.8 g; sodium chloride, 740 g; potassium sulphate; 466 g; magnesium oxide, 240 g; ferric citrate, 60.6 g; zinc carbonate, 16.5 g; manganese carbonate: 6.3 g; copper carbonate, 3 g; potassium iodate, 0.1 g; sodium selenate, anhydrous: 0.103 g, ammonium molybdate.4H_2_O: 0.080 g, sodium metasilicate.9H_2_O: 14.5 g; chromium potassium sulfate.12H_2_O: 2.750 g, lithium chloride: 0.174 g, boric acid, 0.815 g; sodium fluoride, 0.635 g; nickel carbonate, 0.318 g; ammonium vanadate, 0.066 g; powdered sugar, 2210 g.

### Experimental design and sample collections

**Fig. 1.**
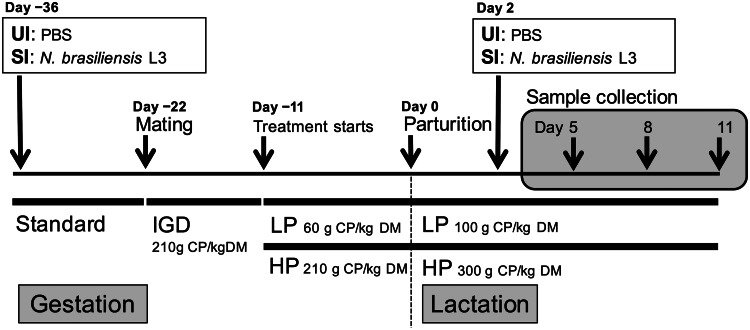
Feeding and infection protocol. Dams were fed *ad libitum*. *n* = 7–9 for secondary infected (SI) dams for each endpoints and *n* = 3 for uninfected dams (UI) dams. IGD, initial gestation diet; LP, low protein; HP, high protein; CP, crude protein; DM, dry matter.

SI groups were euthanized on day 5, 8 and 11 (day 3, 6 and 9 post-secondary infection, respectively), which aimed to provide information on the temporal effects of dietary protein supplementation on maternal performances and immune responses (Fig. 1). UI groups were euthanized on day 5 to provide baseline values for the immune responses measured. On sampling days, dams were sedated through increasing CO_2_ inhalation, and then euthanized by CO_2_ asphyxiation. Pups were euthanized by cervical dislocation. Final total number of rats used in this experiment was 53; five rats had to be removed from the experiment either because they did not conceive, or culled for ethical reasons, such as pupping trauma. Consequently, total sample size for this experiment for each feeding treatment was *n* = 3 for the UI group and *n* = 7 to 9 for the SI group on each endpoint.

### Measurements

#### Performance and parasitology

Pup number was standardized to 10 in all litters on day 1, and pups were bulk-weighed and counted daily until the endpoint. Dams were weighed daily throughout the experiment. DM intake was assessed daily by weighing food offered and refused. On sampling, the small intestine was removed, cut open, placed in saline and incubated for 30 min at 30°C to allow worm recovery for the determination of worm numbers. Colon contents were also recovered from the SI group and colon egg counts (CEC) were performed with the same method previously described for FEC during the primary infection.

#### Histology

At post-mortem, lung and small intestine samples were collected for histology. The left lung was excised and slowly perfused with 4 mL of 10% formaldehyde *via* the main bronchus. A sample from the small intestine was taken 25 cm from the pylorus. Both samples were fixed in 10% formaldehyde, embedded in paraffin and cut into sections; lung samples were stained with haematoxylin and eosin and intestinal samples with periodic acid-Schiff (PAS) for goblet cell quantification. To quantify the destruction of alveolar walls in the lung, ten non-overlapping ×40 magnification images were obtained for each lung to calculate the mean linear intercept (Lmi; Thurlbeck, 1967). Six horizontal lines were drawn across each image using Image J software (Schneider *et al*., 2012) and alveolar wall intercepts were counted per line, and the length of each line was then divided by the number of intercepts to calculate the Lmi value (Sutherland *et al*., 2014). To quantify goblet cells in the intestine, ten villi were randomly selected from one section per animal. Villus surface length was measured, and PAS-positive cells were counted using Image J software. Goblet cell number was expressed as the mean number of goblet cells/villus surface length (*μ*m) (Sakamoto *et al*., 2000; Golder *et al*., 2011).

#### RNA extraction/cDNA synthesis

At post-mortem, samples from the right lung and the small intestine taken at 25 cm distance from the pylorus were fixed in RNA later (Sigma, UK). RNA extraction was performed using Qiagen RNA extraction kit (Qiagen, UK) following the manufacturer's guidelines. cDNA synthesis was conducted using Thermo Scientific Verso™ cDNA synthesis kits (Thermo Scientific, UK) following the manufacturer's guidelines.

#### Real-time quantitative PCR

Primers were selected either from previously published papers or designed with the online Primer Blast software (http://www.ncbi.nlm.nih.gov/tools/primer-blast/). Designed primers were tested *in silico* in Primer stats (http://www.bioinformatics.org/sms2/pcr_primer_stats.html) and mFold (http://mfold.rna.albany.edu/?q=mfold). PCR was performed on a MX300P QPCR system (Agilent Technologies, UK). One *μ*L of cDNA template was added to 20 *μ*L reaction containing 10 *μ*L of Brilliant Ultra-Fast 2 × SYBR^®^ green QPCR Master Mix (Agilent Technologies), 0.3 *μ*L of reference dye, and 0.5 *μ*L of each forward and reverse primer (Supplementary Table S1, Invitrogen, UK). No-template controls were prepared by adding 1 *μ*L of nuclease-free water instead of cDNA template. The thermal profile of the reaction included: 95°C for 3 min, 40 cycles of 95°C for 20 s, annealing at 60°C for 20 s. Real-time PCR was performed with a melting curve analysis to establish the purity of each amplified product. All PCR products were sequenced to verify that intended target products were amplified. Relative mRNA levels were calculated for genes of target using an included standard curve for each individual gene and values normalized to geometric mean of two reference genes selected by the *Rattus norvegicus* 6 gene geNorm kit (PrimerDesign Ltd, UK), *Ywhaz* and *Actb*.

#### Flow cytometry analysis

T helper cell, cytotoxic T cell, B cell and macrophage populations of the spleen and MLN were assessed using CD4^+^, CD8^+^, CD45R^+^ and CD68^+^ antibodies respectively. Cell suspensions of collected spleen and MLN from all the groups were prepared. Following the lysis of red blood cells, the total number of remaining cells was counted by Cellometer Vision^®^ (Nexcelom Bioscience, USA). Cell number was adjusted to 4 × 10^6^ cells per well to 96-well plates and washed with Dulbecco's PBS. Cells were incubated with LIVE/DEAD^®^ stain (Invitrogen) for 10 min at room temperature, and blocked with Fc*γ* Blocker (550270, BD Biosciences, UK) for 15 min on ice. After blocking, cells were incubated with the following monoclonal antibodies, (i) PE-Cy7 conjugated anti-rat CD4 (Clone OX-35, 561578, BD Biosciences), (ii) V450 conjugated anti-rat CD8a (Clone OX-8, 561614, BD Biosciences) and (iii) FITC conjugated anti-rat CD45R/B220 (Clone HIS24, 561876, BD Biosciences) antibodies for 30 min on ice. Cells were washed, incubated with 2% paraformaldehyde for 10 min and stored in FACS buffer (0.5% BSA, 2 mm EDTA and PBS) until the analysis. For the splenic CD68 staining, cells were washed and incubated with Perm buffer for 20 min, and then with Alexa Fluor^®^ 647 anti-rat CD68 (Clone ED1, MCA341A647, Bio-Rad Laboratories, UK) antibody and corresponding IgG isotype control (MCA1209A647, Bio-Rad Laboratories) for 30 min on ice. Cells were re-suspended in 100 *μ*L FACS buffer and analysed using BD FACS Canto™II (BD Biosciences). Collected data were analysed using FlowJo software version 8.7 (Tree Star Inc., Ashland, OR, USA). Gating strategies are shown in Supplementary Fig. S1. Absolute cell numbers were calculated by multiplying the percentage of each cell type with the total cell number of the organ.

### Statistical analysis

Immunological parameters, including histological variables (Lmi value and goblet cell number) and gene expression data, and worm numbers from all SI dams were analysed in a 2 × 3 ANOVA with diet (LP and HP) and three endpoints (days 5, 8 and 11) as factors to test the temporal effects of dietary protein following secondary infection. CEC were analysed on day 11 by one-way ANOVA with diet as a factor. All data were first checked for normality, and a log (*n* + 1) transformation was performed for CEC and worm numbers to stabilize the variance before statistical analysis. Transformed results are reported as backtransformed means with lower and upper limits of 95% confidence intervals. UI dams were used to provide baseline values for responses and shown in the figures as a reference but these values were not included in the statistical model.

Maternal performance data were collected over time to quantify the temporal effects of the nutritional treatments on dam weight, mean pup weight and DM intake data through repeated-measures ANOVA (Littell *et al*., 1998). Dam body weight when mating was confirmed (day −22) was used as a covariate for dam and pup weight analysis. Average food intake in early gestation (days −22 to −11), when all the animals were fed the IGD diet, was used as a covariate for the lactation DM intake analysis.

All statistical analyses were carried out using Prism 7.0 (version 7.0c, GraphPad Software). Values were presented as mean ± s.e. *P* values <0.05 were considered statistically significant.

## Results

### Protein supplementation results in improved performance in dams and pups

Consistent with previous studies, HP dams achieved a significantly heavier body weight compared to LP animals at parturition (LP 289.3 ± 4.3 g, HP 329.5 ± 4.8 g, *P* < 0.0001) through lactation up until day 11 (LP 267.3 ± 6.4 g, HP 340.3 ± 8.1 g, *P* < 0.0001). Pups from HP dams were significantly heavier compared to those from LP dams at birth (LP 4.79 ± 0.12 g, HP 5.85 ± 0.14 g, *P* < 0.0001) through lactation (LP 15.39 ± 0.70 g, HP 23.18 ± 0.88 g, *P* < 0.0001). Average daily pup weight gain was also significantly heavier in HP dams (1.58 ± 0.14 g) compared to LP dams (0.96 ± 0.08 g, *P* = 0.002). Overall DM intake did not differ between LP and HP diet until day 5, but it became significant showing increased intake in HP dams compared to LP dams on day 8 (LP 26.78 ± 1.47 g, HP 39.89 ± 1.57 g, *P* < 0.0001) and day 11 (LP 25.68 ± 2.80 g, HP 53.26 ± 3.24 g, *P* < 0.0001) (Table 2).

**Table 2.** Results for maternal performances during lactation

	Dam weight (g)			Pup weight (g)			DM intake (g)		
Day	LP	HP	*P*	LP	HP	*P*	LP	HP	*P*
0^a^	289.3 ± 4.3	329.5 ± 4.8	<0.001	4.79 ± 0.12	5.85 ± 0.14	<0.001	–	–	–
2^b^	291.6 ± 4.0	325.2 ± 4.5	<0.001	5.96 ± 0.18	7.72 ± 0.21	<0.001	20.26 ± 1.04	20.80 ± 1.13	= 0.741
5	287.1 ± 3.9	319.7 ± 4.4	<0.001	8.73 ± 0.27	11.98 ± 0.31	<0.001	24.59 ± 1.26	27.89 ± 1.38	= 0.087
8	278.2 ± 3.7	325.8 ± 4.3	<0.001	11.92 ± 0.45	17.34 ± 0.52	<0.001	26.78 ± 1.47	39.89 ± 1.57	<0.001
11	267.3 ± 6.4	340.3 ± 8.1	<0.001	15.39 ± 0.70	23.18 ± 0.88	<0.001	25.68 ± 2.80	53.26 ± 3.24	<0.001
Average daily gain	−2.00 ± 0.71	0.98 ± 1.56	= 0.103	0.96 ± 0.08	1.58 ± 0.14	= 0.002	1.21 ± 0.77	3.74 ± 0.93	= 0.052

DM, dry matter; IGD, initial gestation diet; LP, low protein; HP, high protein.

a Day 0: Day of parturition. Dams were weighed after parturition.

b Day 2: Dams received a secondary infection with 1600 L3.

### Protein supplementation upregulates the expression of genes related to Th2 immunity in the damaged lung

**Fig. 2.**
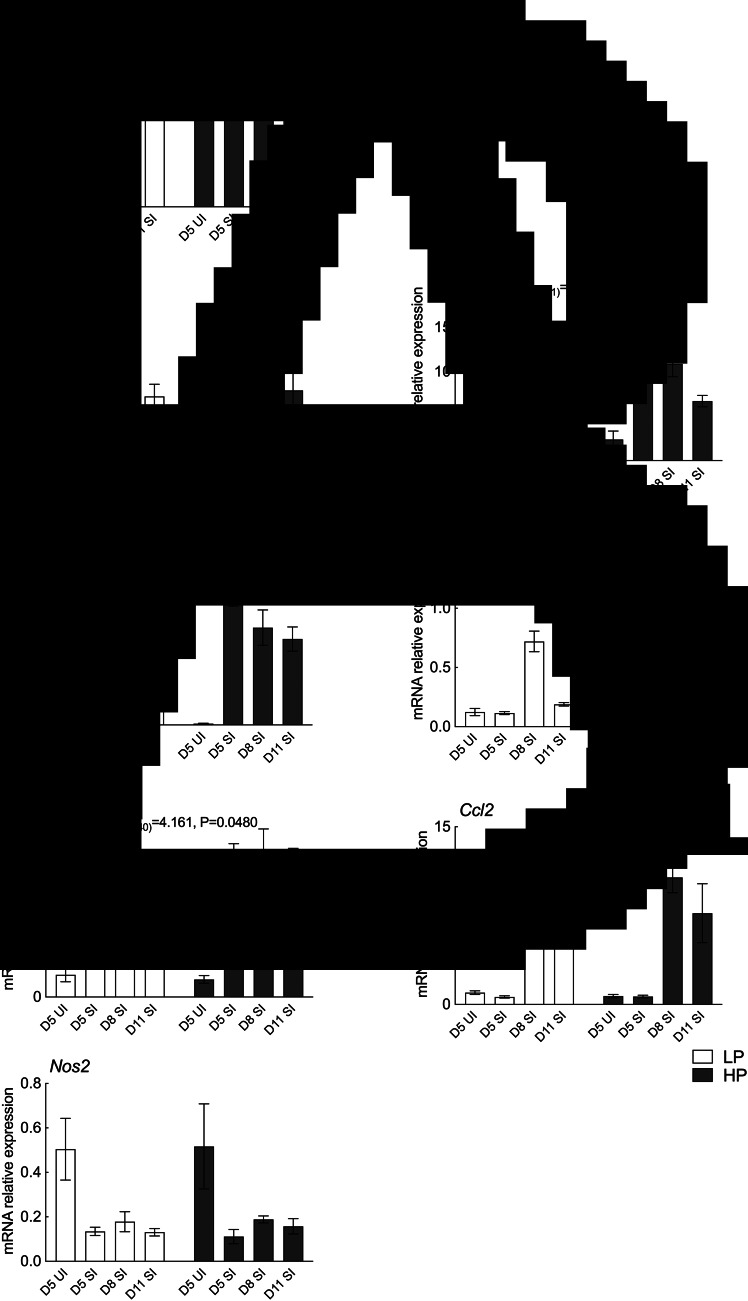
Histology and gene expression results for the lung. Samples were analysed in 2 (diet) × 3 (endpoint) factorial ANOVA. UI groups were included for baseline references. Standard errors are shown by vertical bars. LP, low protein; HP, high protein. (A) Average mean linear intercepts. (B) Real-time quantitative PCR gene expression analysis. Values were normalized using reference genes, *Actb* and *Ywhaz*.

Infiltration of inflammatory cells and haemorrhage in the lung airspace and around the blood vessels were observed on day 5 in SI dams, which were largely cleared by day 11 (data not shown). Lmi values were used to assess lung damage and these were highest on day 11 for both LP (57.81 ± 5.29 *μ*m) and HP dams (57.83 ± 2.67 *μ*m) with no effect of dietary treatments (Fig. 2A). However, dietary treatments did affect gene expression profiles in the inflamed/damaged lung. There was a significant main effect of dietary treatment on the expression of *Retnla* where expression was upregulated in HP dams compared to LP dams (*P* < 0.001). Likewise, a significant main effect of dietary treatment was observed for *Il13* (*P* = 0.0194) and *Arg1* (*P* = 0.0385) where HP dams had increased expression compared to LP dams. HP dams also had increased expression of *Mmp12* (*P* = 0.0480), an elastin degrading enzyme which plays a role in pulmonary emphysema development, compared to LP dams. Neither nitric oxide synthase (*Nos2*), a marker for classically activated macrophages, nor chemokine ligand 2 (*Ccl2*), an essential cytokine for monocyte recruitment to inflamed lungs, were affected by dietary treatments. No diet × endpoint interaction effect was observed for all the genes examined in the lung (Fig. 2B).

### Protein supplementation is associated with reduced number of goblet cells and shifts in goblet cell-associated transcriptional activities in the small intestine

**Fig. 3.**
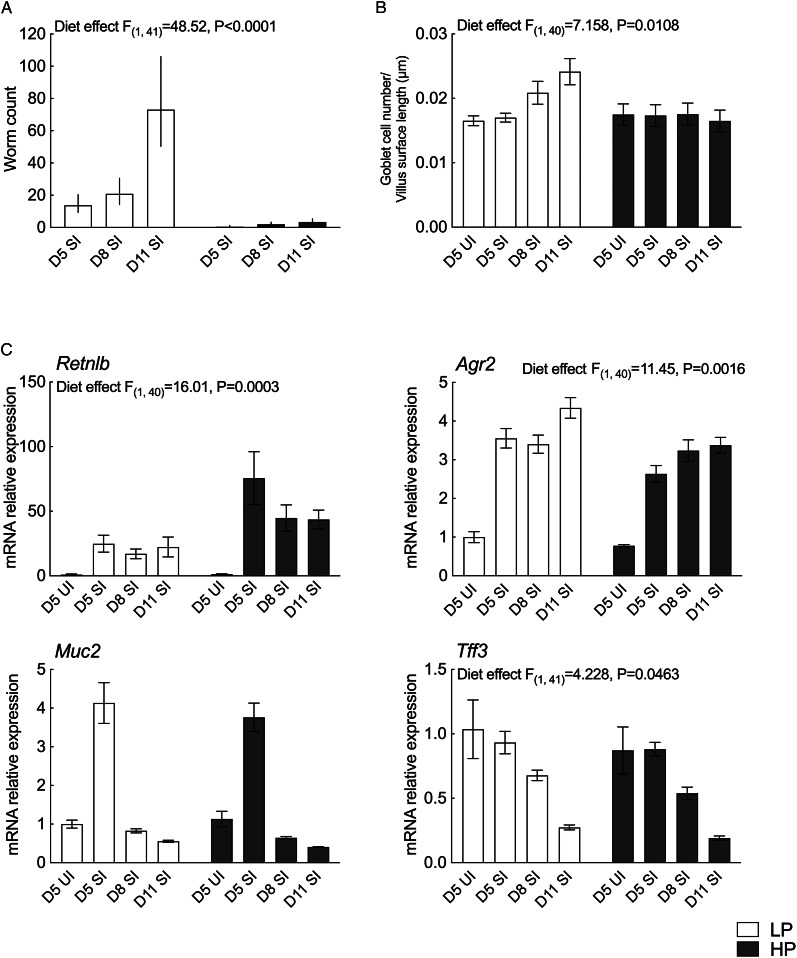
Histology and gene expression results for the intestine. Samples were analysed in 2 (diet) × 3 (endpoint) factorial ANOVA. UI groups were included for baseline references. LP, low protein; HP, high protein. (A) Worm number for all endpoints. Data were Log (n + 1) transformed and values are shown as backtransformed mean with backtransformed lower and upper limits of transformed error bars. (B) Goblet cell counts. Standard errors are shown by vertical bars. (C) Real-time quantitative PCR gene expression analysis of the intestine. Values were normalized using reference genes, *Actb* and *Ywhaz*. Standard errors are shown by vertical bars.

HP dams carried significantly fewer worm numbers than LP dams on all endpoints; mean worm number through the experiment was 51.1 (range 0–276) for LP dams and 2.3 for HP dams (range 0–12; Fig. 3A). LP dams had significantly higher goblet cell numbers/villus surface length (0.024 ± 0.002 *μ*m) than HP dams (0.017 ± 0.002 *μ*m, main effect of diet *P* = 0.0108; Fig. 3B). Similarly, the expression of genes associated with goblet cells and mucus production, anterior gradient homologue (*Agr2*) and trefoil factor family 3 (*Tff3*), was upregulated in LP dams compared to HP dams (main effect of diet, *P* = 0.0016 and *P* = 0.0463, respectively, Fig. 3C). A significant main effect of diet (*P* = 0.0003) was also evident for resistin-like beta (*Retnlb*) expression, a goblet cell peptide regulated by Th2 cytokines, where significant increase was observed in HP dams compared to LP dams (Fig. 3C). mRNA expressions of mucin 2 (*Muc2*), a mucin secreted by goblet cells, was not affected by dietary treatments. No diet × endpoint interaction effect was observed on all the genes examined in the intestine.

### Protein supplementation increased cell number, CD8^+^ and CD68^+^ cell population in the spleen, and CD4^+^ and CD8^+^ cell population in the MLN of lactating rats

**Fig. 4.**
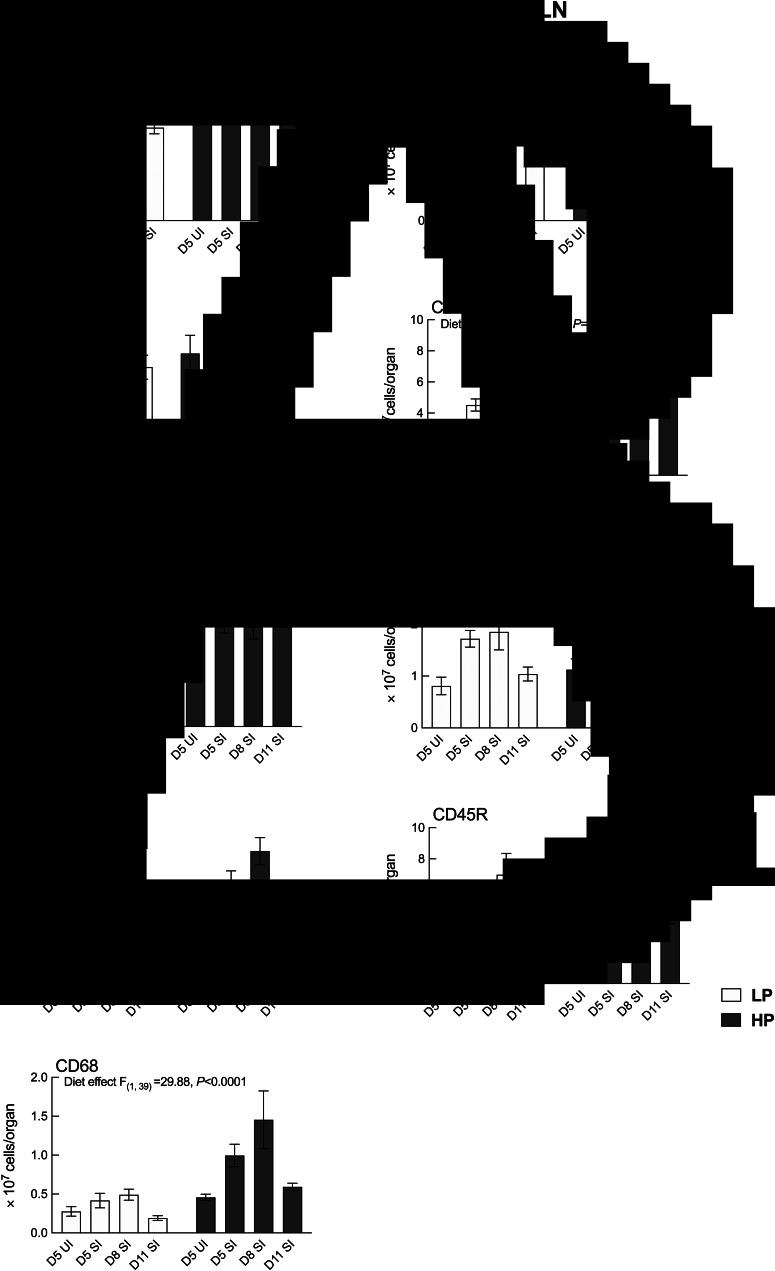
Results for cell number and CD4^+^, CD8^+^, CD45R^+^ and CD68^+^ population in the spleen (A) and the MLN (B). Samples were analysed in 2 (diet) × 3 (endpoint) factorial ANOVA. UI groups were included for baseline references and standard errors are shown by vertical bars. LP, low protein; HP, high protein.

Significant main effect of the diet was observed for total cell number, CD8^+^ and CD68^+^ population in the spleen (*P* = 0.002, *P* = 0.0007 and *P* < 0.0001, respectively), where HP dams showed higher recovery compared to LP dams (Fig. 4A). Likewise, significant main effect of the diet was observed for CD4^+^ and CD8 population (*P* = 0.0341 and *P* = 0.0024, respectively), where HP dams showed higher recovery compared to LP dams (Fig. 4B). No diet × endpoint interaction effect was observed for cell number or cell populations in the spleen or the MLN.

## Discussion

Protein supplementation consistently improves resistance to *N. brasiliensis* in periparturient rats as demonstrated by FEC, worm counts and intestinal immune response (Houdijk *et al*., 2005; Jones *et al*., 2009; Athanasiadou *et al*., 2011), consistent with the reduced worm burden in HP dams *vs* LP dams in this study. Here we show that protein supplementation enhances the expression of Th2 immunity in the lung, which may have contributed to the resolution of the local inflammation and negatively impacted the survival of the parasites. We also show that the changes in gene expression at the sites where parasites are present, such as the lung and small intestine, are accompanied with a shift in splenic and MLN immune cell populations.

Protein supplementation resulted in enhanced Th2 immune response in the lung, which was manifested with increased expression of *Il13*, *Retnla* and *Arg1*. The lung is a crucial site in priming protective immunity against *N. brasiliensis* where lung-resident CD4^+^ T cells elicit IL-4R*α* dependent protection against secondary infection (Harvie *et al*., 2010; Thawer *et al*., 2014). IL-13, a major Th2 cytokine which is signalling *via* the IL-4R*α*, is known to activate AAMs and alter epithelial cell phenotypes (Urban *et al*., 1998; Herbert *et al*., 2009; Gordon and Martinez, 2010; Gerbe *et al*., 2016). In addition, recent evidence has shown that IL-13 is crucial in limiting airway haemorrhaging and tissue injury following acute lung damage (Chenery *et al*., 2021). RELM*α* is induced by chitinase-like protein Ym1, a key player in regulating Th2 immune response and enhances lung repair following *N. brasiliensis* larval migration (Sutherland *et al*., 2018). Similarly, Arginase 1 also regulates Th2 immune response and suppresses excessive lung inflammation (Pesce *et al*., 2009). These data collectively support that protein supplementation not only triggered an enhanced Th2 immune response but it may have contributed to the efficient resolution of inflammation in the lung caused by the invading parasites.

Although the Lmi score showed no difference in the destruction of alveolar walls in the lung in rats offered LP and HP, expression of *Mmp12*, a gene encoding a macrophage-specific elastin-degrading enzyme, was higher in the lungs of HP compared to LP rats. Mmp12 has been associated with pathological conditions and is reported to be a major contributing factor to the development of emphysema (Houghton, 2015; Wynn and Vannella, 2016). *Nippostrongylus brasilensis* infection has been reported to cause emphysema with the progression of enlarged airspace overtime and increased expression of *Mmp12* (Marsland *et al*., 2008). The role of HP diet in the resolution of local inflammation on the one hand, and emphysema on the other requires further investigation; the possibility that protein supplementation may not overcome long-term detrimental effects on the lung of parasitized animals cannot be excluded.

This study showed an association between protein supplementation and reduced worm burden as early as day 5 (day 3 post-secondary infection); previous studies have shown an impact of protein supplementation from day 11 (day 9 post-secondary infection) onwards (Jones *et al*., 2009; Athanasiadou *et al*., 2011). The early impact on worm burden observed here could be associated with the earlier implementation of the nutritional treatments than previous experiments, which started from the second half of gestation. Our data collectively indicate that the enhanced Th2 response in the lung of HP dams, as observed with increased expression of *Il13*, *Retnla*, and *Arg1*, may have reduced larvae number in the lung and consequently reduce the number of parasites reaching the intestine. Previous evidence has shown significantly increased cellular inflammation and mucus production in the lungs of *N. brasiliensis* reinfected mice, which suggests worms can be trapped and cleared within lung tissue (Harvie *et al*., 2010). In support of this hypothesis, HP dams showed goblet cell number similar to UI control, which may be indicative of low number of viable parasites reaching the small intestine in the HP dams. The presence of worms in the intestine accelerates the proliferation of goblet cells and increased the secretion of the mucus to effectively expel worms from the intestine (Miller and Nawa, 1979; Khan *et al*., 2001). Indeed, higher worm burden in LP dams here may have triggered an increase in mucus secretion which was reflected in goblet cell number and also an increase in *Agr2* expression (Park et al., 2009). On the other hand, an increase in the expression of *Retnlb* was observed in HP dams. RELM*β* can be induced directly by bacterial products and is profoundly influenced by host metabolism (Pine *et al*., 2018). Secretion of RELM*β* may therefore be directly modulated by the HP diet, consistent with the sensitivity of RELM*β* expression to dietary components in the colon of C57BL mice (Hildebrandt *et al*., 2009) or changes to the microbiome caused by dietary changes. Alternatively, the increased expression of RELM*β* in HP rats may reflect cross-mucosal immunity between the lung and the intestine, as reported for a strictly enteric nematode in the gut that elicits innate IL-13-driven protection at distal mucosal sites, including the lung (Campbell *et al*., 2019). Indeed, *Retnlb* expression in the intestine of HP-fed dams here follows the expression pattern of *Il13* and *Retnla* in the lung. Secretion of RELM*β* from the goblet cells has been shown to impair the motility of *N. brasiliensis*, contributing to worm expulsion (Herbert *et al*., 2009); thus, upregulation of *Retnlb* expression in HP-fed dams in the present experiment may have contributed to larger or earlier reduction of viable worms in the intestine. *Retnlb* expression remained lower in LP dams compared to HP dams and worm numbers continued to increase until day 11.

The changes in the lung and small intestine reported above were accompanied with a shift in splenic and MLN immune cell populations. Protein malnutrition has been associated with a reduction of absolute numbers in splenic T-cell and B-cell populations in mice, irrespective of the presence of a pathogenic stimulus (Mello *et al*., 2014). LP dams in our study also showed a reduced number of CD4^+^ and CD8^+^ cells in the MLN and CD8^+^ cells in the spleen compared to HP dams. Although T-cell populations were sensitive to dietary treatment, B cells, as indicated by the number of CD45R^+^ (B220^+^) cells, were not affected by the diet. B-cell populations in secondary lymphoid organ are reported to promote protective immunity against *N. brasiliensis* re-infection (Horsnell *et al*., 2013), but the effect of dietary protein on immune cells may vary depending on factors such as cell types and developmental stages, which is yet to be elucidated.

Splenic macrophage population marked by CD68^+^ was increased in HP compared to LP dams following secondary infection of *N. brasiliensis*. Protein and protein-energy malnutrition have been reported to result in a higher level of macrophage apoptosis and in the reduction of phagocytic/fungicidal activities, which shows that undernourishment can impair both the macrophage number and immune functions (Rivadeneira *et al*., 2001; Santos *et al*., 2016). Increased cell death may be one of the reasons for a lower number of macrophages in our LP dams as feeding treatment here created a sustained deficiency in dietary protein. It is also possible that the production of cytokines which stimulates macrophage activation was reduced in LP dams. We have previously demonstrated downregulated expression of *Il4* in the spleen in protein-deficient animals during the gestation period (Masuda *et al*., 2018). As IL-4 is an important macrophage proliferation/survival cytokine during helminth infection (Jenkins *et al*., 2013), LP diet may have reduced IL-4 production in the spleen, which has limited the macrophage proliferation and activation in the spleen.

In conclusion, we have demonstrated here that dietary protein supplementation during gestation and lactation results in low parasite burden and this is associated with changes at gene and cellular level in rats infected with *N. brasiliensis*. Our data support the view that some of the effects are mediated directly *via* the protein supplementation (or scarcity), whereas others are mediated *via* lower parasite burden in the protein-supplemented animals. We have shown that protein supplementation during the early phase of gestation is important for efficient immune response against parasite infection while maintaining healthy pregnancy. Such information is expected to define strategic utilization of nutrient supply and to complement existing sustainable parasite control strategies in mammals.

## Supplementary material

For supplementary material accompanying this paper visit https://doi.org/10.1017/S0031182021001876. click here to view supplementary material
