# Supplementary material for: Dietary protein supplementation results in molecular and cellular changes related to T helper type 2 immunity in the lung and small intestine in lactating rats re-infected with Nippostrongylus brasiliensis
Source: Parasitology. 2021 Nov 3;149(3):337–46. doi: 10.1017/S0031182021001876 (PMC10090644; doi:10.1017/S0031182021001876)
Supplement: Supplementary file 1 [file S0031182021001876sup.zip › S0031182021001876sup001.docx]

MLN

Spleen

**Supplementary Figure 1.** Gating strategies for MLN and splenic cells. Live leukocytes were selected from the forward/side scatter plot and gating negative Live/Dead stain population. Then viable cells were sorted based on surface staining of CD4 and CD8a. CD45R population was gated on CD4, CD8 double negative population. Splenic CD68 positive cells were gated on live leukocyte population.
