# Supplementary material for: Dietary protein supplementation results in molecular and cellular changes related to T helper type 2 immunity in the lung and small intestine in lactating rats re-infected with Nippostrongylus brasiliensis
Source: Parasitology. 2021 Nov 3;149(3):337–46. doi: 10.1017/S0031182021001876 (PMC10090644; doi:10.1017/S0031182021001876)
Supplement: Supplementary file 1 [file S0031182021001876sup.zip › S0031182021001876sup002.docx]

**Supplementary Table 1**. Primers used for real time quantitative PCR.

| **Gene name** | **Accession number** | **Forward** | **Reverse** | **Amplicon length(bp)** | **Reference** |
| --- | --- | --- | --- | --- | --- |
| *Il4* | NM_201270.1 | CAAGGAACACCACGGAGAA | TTCAGACCGCTGACACCTC | 151 | Lively and Schlichter, 2012 |
| *Il13* | NM_053828 | CTCAGGGAGCTTATCGAGGA | CGAGGCCTTTTGGTTACAGA | 201 | Athanasiadou *et al*., 2011 |
| *Arg1* | NM_017134 | TGGAACGAAACGGGAAGGTA | CTGTGATGCCCCAGATGACTT | 101 | Gebel *et al*., 2006 |
| *Retnla* | NM_053333 | CAGCTGATGGTCCCAGTGAAT | TCCCAAGATCCACAGGCAAA | 212 |  |
| *Mmp12* | NM_053963.2 | CTCCCATGAACGAGAGCGAA | GGTGTCCAGTTGCCCAGTTA | 170 |  |
| *Nos2* | NM_012611 | GAAACTTCTCAGCCACCTTGG | ACAAGACCCAAGCCTGAGGA | 76 |  |
| *Ccl2* | NM_031530.1 | CAGCAGCAGGTGTCCCAAAGAAGC | AGGTGGTTGTGGAAAAGAGAGTGGA | 242 |  |
| *Retnlb* | NM_001024281 | ACGCAGTGCTCCTTTGAGTC | GACAACCATCCCAACAGGACA | 135 |  |
| *Agr2* | NM_053392 | CAAAGGACTCTCGACCCAAA | ACTGCTCTGCCAATTTCTGG | 213 | Athanasiadou *et al*., 2011 |
| *Muc2* | XM_008760048.1/  XM_008774808.1 | GGGACACCATGTGCCCTTTA | GACCACATACATTGCCCTTGT | 143 |  |
| *Tff3* | NM_013042 | ATGGAGACCAGAGCCTTCTG | TGGGATGCTGGAGTCAAAACA | 195 | Yamauchi *et al*., 2006 |
| *Actb* | NM_031144 | CGTTGACATCCGTAAAGACC | TAGAGCCACCAATCCACAC | 176 | Athanasiadou et al., 2011 |
| *Ywhaz*  (PrimerDesign Ltd, UK)* | NM_013011 | - | - | 187 |  |

* PrimerDesign Ltd, UK owns the proprietary rights for the primer sequence as it is part of the geNorm Kit and cannot be referenced here.

References

**Lively S and Schlichter LC** (2012) Age-related comparisons of evolution of the inflammatory response after intracerebral hemorrhage in rats. *Translational Stroke Research* **3**, 132-146.

**Athanasiadou S, Jones LA, Burgess ST, Kyriazakis I, Pemberton AD, Houdijk JG and Huntley JF** (2011) Genome-wide transcriptomic analysis of intestinal tissue to assess the impact of nutrition and a secondary nematode challenge in lactating rats. *PloS One* **6**.

**Gebel S, Gerstmayer B, Kuhl P, Borlak J, Meurrens K and Müller T** (2006) The kinetics of transcriptomic changes induced by cigarette smoke in rat lungs reveals a specific program of defense, inflammation, and circadian clock gene expression. *Toxicological Sciences* **93**, 422-431.

**Yamauchi J, Kawai Y, Yamada M, Uchikawa R, Tegoshi T and Arizono N** (2006) Altered expression of goblet cell-and mucin glycosylation-related genes in the intestinal epithelium during infection with the nematode *Nippostrongylus brasiliensis* in rat. *APMIS* **114**, 270-278.
